# Effects of sugary drinks, coffee, tea and fruit juice on incidence rate, mortality and cardiovascular complications of type2 diabetes patients: a systematic review and meta-analysis

**DOI:** 10.1007/s40200-024-01396-5

**Published:** 2024-04-08

**Authors:** Ping Ding, Wei Yue, Xu Wang, Yuqing Zhang, Yuxiang Liu, Xiaofeng Guo

**Affiliations:** 1 Department of Critical Care Medicine, No. 988 hospital of The PLA Joint Logistic Support Force (PLAJLSF), Zhengzhou, 450000 China; 2 School of Clinical Medicine, Zhengzhou University, Zhengzhou, 450000 China

**Keywords:** Beverages, Sugar-sweetened beverages, Artificially-sweetened beverages, Type 2 diabetes mellitus, Meta-analysis

## Abstract

**Aims:**

Despite more and more studies indicate that beverages play an important role in type 2 diabetes mellitus(T2DM), the efficacy of intaking different beverages for T2DM has not been clearly stated in one article. The meta-analysis was performed, which aims to assess the effects of beverages on mortality and cardiovascular complications in patients with type 2 diabetes and the incidence of T2DM.

**Method:**

PubMed, Embase, Web of Science and Cochrane Library databases were search up to March, 2023 to identify relevant studies, including studies researching beverage consumption, the incidence and mortality of T2DM and incidence of cardiovascular disease, a kind of complication of T2DM. The way to explore the source of heterogeneity is performing subgroup analyses and sensitivity analyses. Funnel plots and Egger’s regression test were performed to assess publication bias. The Hazard ratio (HR) and 95% confidence intervals (95% CIs) were used to analysis the results. Fifteen observational studies were included in our meta-analysis.

**Results:**

Fifteen eligible articles were included sugar-sweetened beverages(SSB) consumption increased the mortality and incidence of T2DM ( Hazard ratio (HR), 1.20; 95% confidence interval (CI), 1.05–1.38; *P* = 0.01 and HR, 1.15; 95% CI,1.06–1.24; *P* = 0.001), respectively. Artificially-sweetened beverages (ASB) consumption was not associated with the mortality and incidence of T2DM (HR,0.96;95%CI, 0.86–1.07; *P* = 0.464 and HR, 1.15; 95% CI,1.05–1.26; *P* = 0.003), respectively. Fruit juice consumption increased the incidence of T2DM (HR,1.08;95%CI,1.02–1.14, *P* = 0.296).

Tea or coffee consumption can reduce the incidence of T2DM (HR, 0.89; 95%CI,0.81–0.98; *P* = 0.016). Tea or coffee consumption was associated with a lower risk of mortality of T2DM (HR,0.84; 95% Cl, 0.75–0.94; *P* = 0.002 and HR,0.75; 95% CI, 0.65–0.87; *P* < 0.001), respectively. Additionally, beverage consumption was not associated with cardiovascular disease in T2DM patients (HR,1.03; 95% Cl, 0.82–1.30, *P* > 0.05).

**Conclusions:**

High consumption of SSBs led to a higher risk and mortality of T2DM, while high consumption of coffee or tea showed significant associations with a lower risk of the incidence and mortality of T2DM.

**Supplementary Information:**

The online version contains supplementary material available at 10.1007/s40200-024-01396-5.

## Introduction

Type 2 diabetes mellitus(T2DM) is one of the most serious public health concerns all over the world and its mortality and incidence is increasing. Many studies have focused on exploring the association between T2DM and beverages consumption. Beverages, a previous study suggests that, account for a significant portion of our diet. This meta-analysis focuses on beverages including coffee, tea, sugar-sweetened beverages (SSBs), artificially-sweetened beverages (ASBs) and natural juices (NJs).

The definition of SSBs is beverages with added caloric sweeteners. Recent large cohorts, followed up for long periods (from 4–20 years), suggested that SSB consumption increased the incidence risk of T2DM [1]. ASBs are concerned to reduce excess energy intake [2], which are defined as all kinds of low-calorie or diet beverages with added artificial sweetener. Previous study [3] has indicated that replacing SSBs with ASBs can possibly reduce the risk of diabetes long-term by positively affecting body weight. There is a study [4] reported that compared with those who did not consume coffee, participants drinking at least 7 cups of coffee per day were half as likely to develop T2DM. Previous study [5] has indicated that replacing SSBs with coffee or tea can possibly reduce the risk of T2DM and the consumption of SSB increased the incidence of T2DM. A recent study [6] reported that the consumption of green tea was associated with a lower risk of incident T2DM and all-cause mortality in patients with diabetes. Additionally, there is a significant association between high consumption of SSBs and a higher risk of T2DM mortality, while the consumption of ASBs, coffee, tea was negatively associated with all-cause mortality [7].

In recent decades, many meta-analyses of observational population-based studies have been published, which focused on the associations between beverages consumption and a range of T2DM outcomes, however, drawing definitive conclusions is difficult due to deficiencies in the study design, varying measurements of dietary beverages consumption, inconsistent findings, and different definitions of exposure. In order to obtain a reliable and comprehensive result, the systematic review and meta-analysis of prospective cohort studies were conducted, which aim is to assess the association between the habitual consumption of different beverages and incidence rate of T2DM, case fatality rate of T2DM and the cardiovascular disease (CVD) complications of T2DM.

## Methods

This systematic review and meta-analysis was written based on the Preferred Reporting Items of Systematic Reviews and Meta-Analysis (PRISMA) statement guidelines [8].

### Search methods

**Fig. 1:**
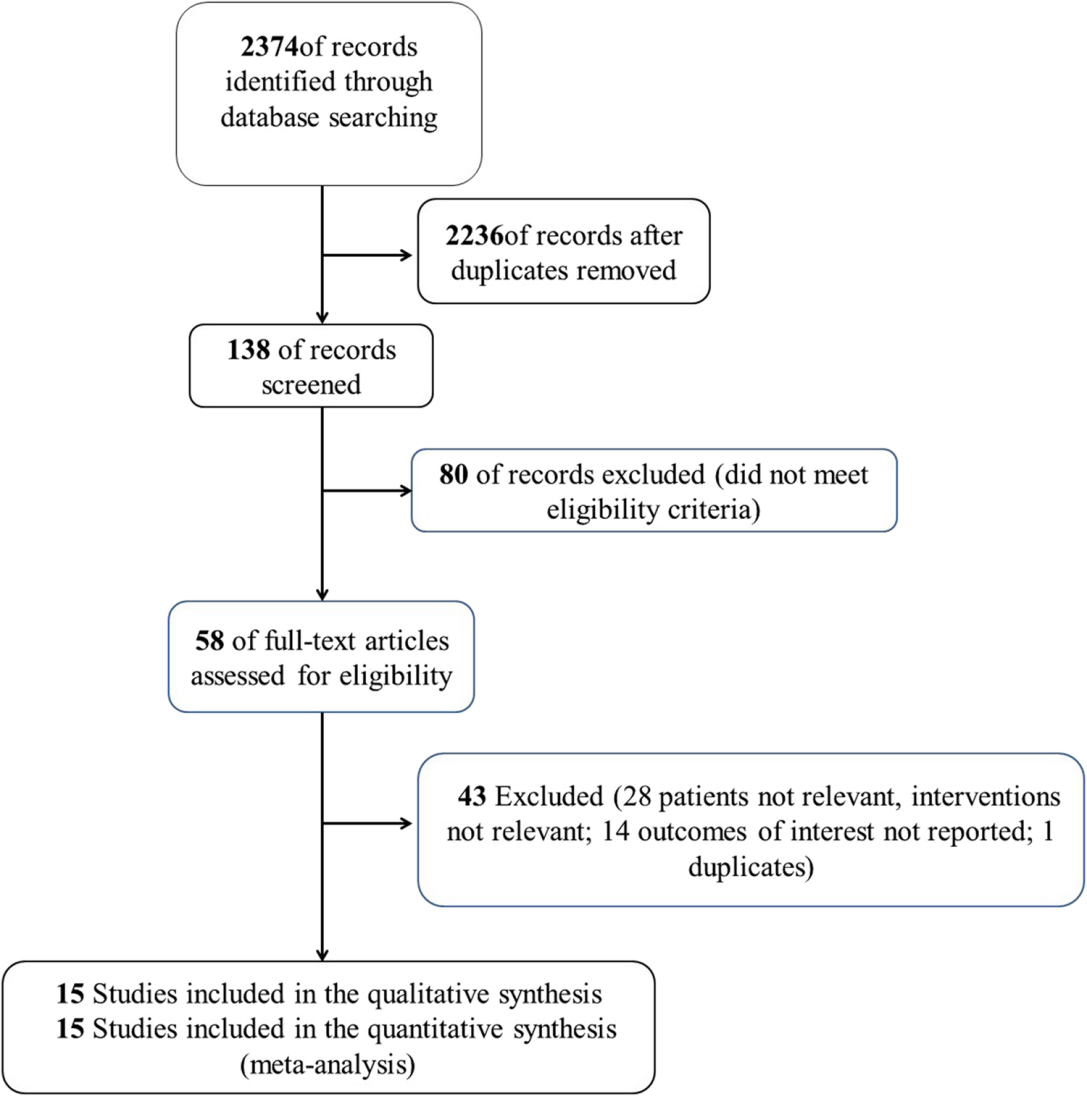
Flowchart of selecting studies for review

The databases searched for this article include PubMed, Embase, Web of science and Cochrane Library databases, and the search authors include Zhang Yuqing, Wang Xu and Liu Yuxiang. The search term is ((type 2 diabetes [Title/Abstract]) OR (type 2 diabetes mellitus[MeSH Terms])) AND ((beverage[MeSH Terms]) OR (beverage[Title/Abstract])), which is consist of main Mesh and non-Mesh terms. The search of relevant literature were searched for relevant studies from inception to March 2023. The MeSH/Emtree and abstract/title keyword were combined to identify the eligible English articles. In addition, a manual search was conducted for the references of the included articles (shown in Fig. 1).

### Study eligibility

Eligibility criteria and exclusion criteria should be identified, which were used to search and screen potential articles. Articles may be eligible, if they fulfill all the following conditions: (1) prospective population-based studies or patients diagnosed with T2DM; (2) the study compared with the use of beverages (including coffee, tea and SBs) with no use of beverages; (3) the study measured and reported the outcomes in terms of mortality of T2DM, incidence of T2DM or incidence of complications; The exclusion criteria were as follows: (1) all criteria were not fulfilled; (2) the study was a case report, an animal study, a review, a conference abstract or an abstract; (3) OR/RR/HR with 95% CIs were not provided or could not be calculated;

### Date extraction and study quality

The selection and searching of potential articles were completed independently by two researchers, based on the inclusion and exclusion criteria, to identify eligible studies. Data extraction was conducted separately by another researchers. Moreover, any disagreements would be decided by the senior researcher to resolve this controversy. Relevant data were collected, which include the year of publication, first author, study title, study type, study period, type of beverage, the efficacy in control and treatment groups, T2DM definition, reported outcomes and so on. The characteristics of the studies included in this meta-analysis included beverage type, follow-up duration range, number of participants, age, sex distribution, beverage intake, and outcome measures (shown in Table 1). Beverage portion sizes and frequency of consumption were different among the included articles, some were not mentioned in the original study, the available data have been extracted into the Table 1. For every abstracted data of articles, two investigators independently assess the risk of bias for this meta-analysis based on The Newcastle–Ottawa Scale (NOS) [9] (shown in Table 2). Articles were thought high quality which received seven or more points. Covariates were composed of eight parts: age and sex, race and socioeconomic status, physical activities, smoking and drinking, body mass index, diet factors, clinical features and other types of beverages. The types of statistical data related to outcomes extracted in these 15 articles were all binary variables, HR values, and 95% confidence intervals.

**Table 1:**
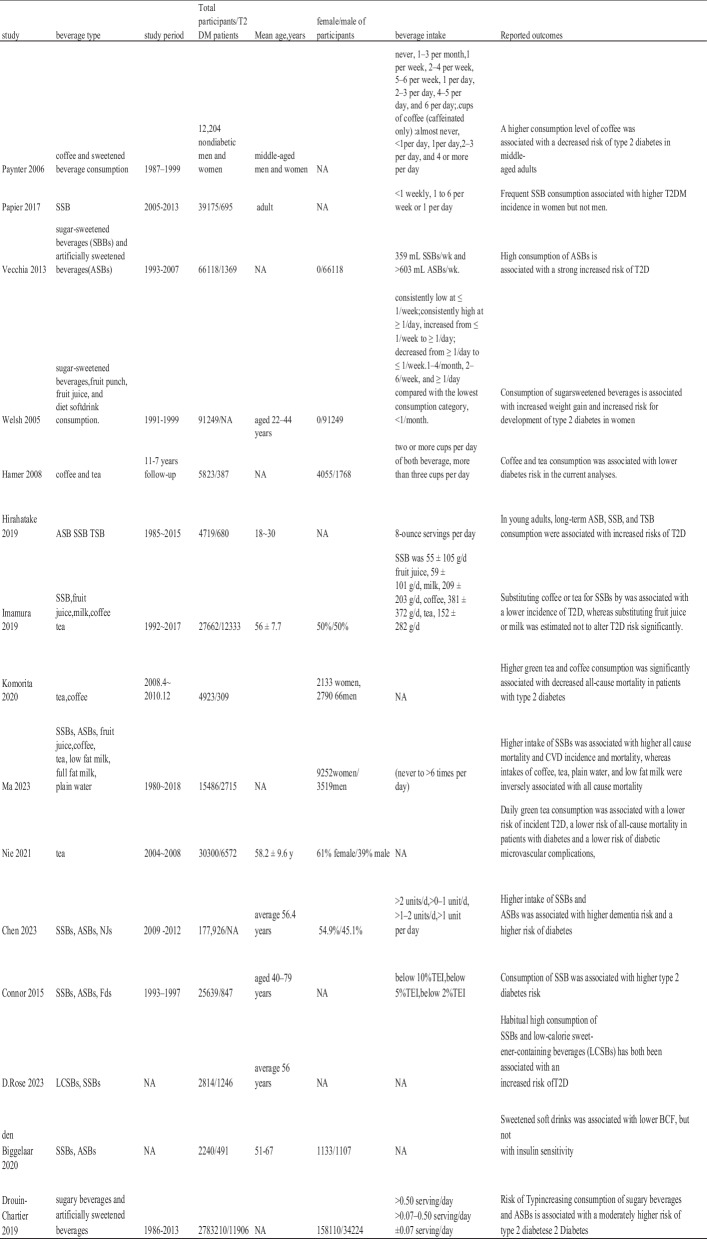
Characteristics of the included studies about the association between beverages and the risk of incidence or prognosis in T2DM patients

Abbreviation

*T2DM* Type 2 of diabetes mellitus; *SSB* Sugar-sweetened beverages; *ASB* Artificially sweetened beverages; *TSB* Total sweetened beverage (combined ASB and SSB); *NJs* Natural juices; *LCSBs* Low-calorie sweetened beverage

**Table 2:**
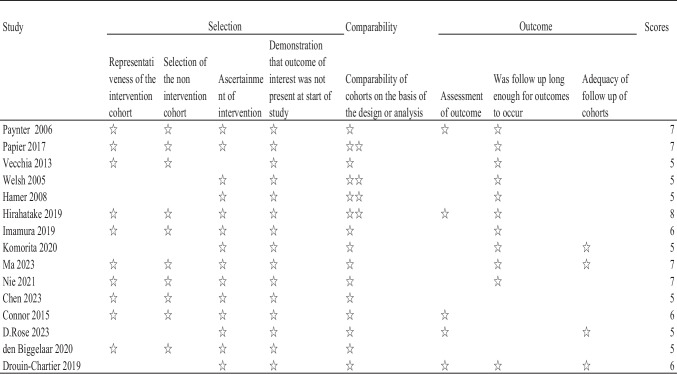
NOS scores of the included studies

### Statistical analysis and publication bias

The aim of forest plots was to synthesize HRs and 95%CI across studies. The I^2^ statistics presented the potential heterogeneity; the pooling outcome was considered to represent non-significant, low, moderate and high heterogeneity respectively, when I^2^ was 0–25%, 26–50%, 51–75% or > 75%. The source of heterogeneity was explored by performing subgroup analysis. The potential source of heterogeneity includes the definition of exposure, grouping criteria, study sites, number of participants, sex ratio, follow-up period, quality of study, baseline age and adjustment of clinical features. Egger’s linearity regression test and Begg’s funnel plot was performed to evaluate the publication bias. For the asymmetry, funnel plots were visually assessed. The aim of Egger’s lire available to assess, sensitivity analysis was performed to evaluate the influence of a single study on the overall effect estimate by sequential stepwise omitnear regression is to confirm the presence of a small size. If sufficient data weting of one study. All analyses were completed by using Stata12.0(College Station, Texas 77845, USA), random effects model and inverted variance method. *P* < 0.05 was considered statistically significant.

## Results

### Selection and identification of studies

Through our search, we initially searched 2486 articles, and 128 remined after removing the literature that did not meet the criteria. After preliminary screening of the title and abstract, we selected 45 articles related to the impact of beverages consumptions on T2DM. According to the above inclusion and exclusion criteria, after the study selection, 15 observational studies [3, 5–7, 10–20] were included in our meta-analysis (shown in Fig. 1). The characteristics of the eligible studies are listed in Table 1. Fifteen observational studies focused on beverages including coffee, tea, sugar-sweetened beverages (SSBs), artificially-sweetened beverages (ASBs) and natural juices (NJs).

### Association of beverages consumptions and all-cause mortality of T2DM patients

**Fig. 2:**
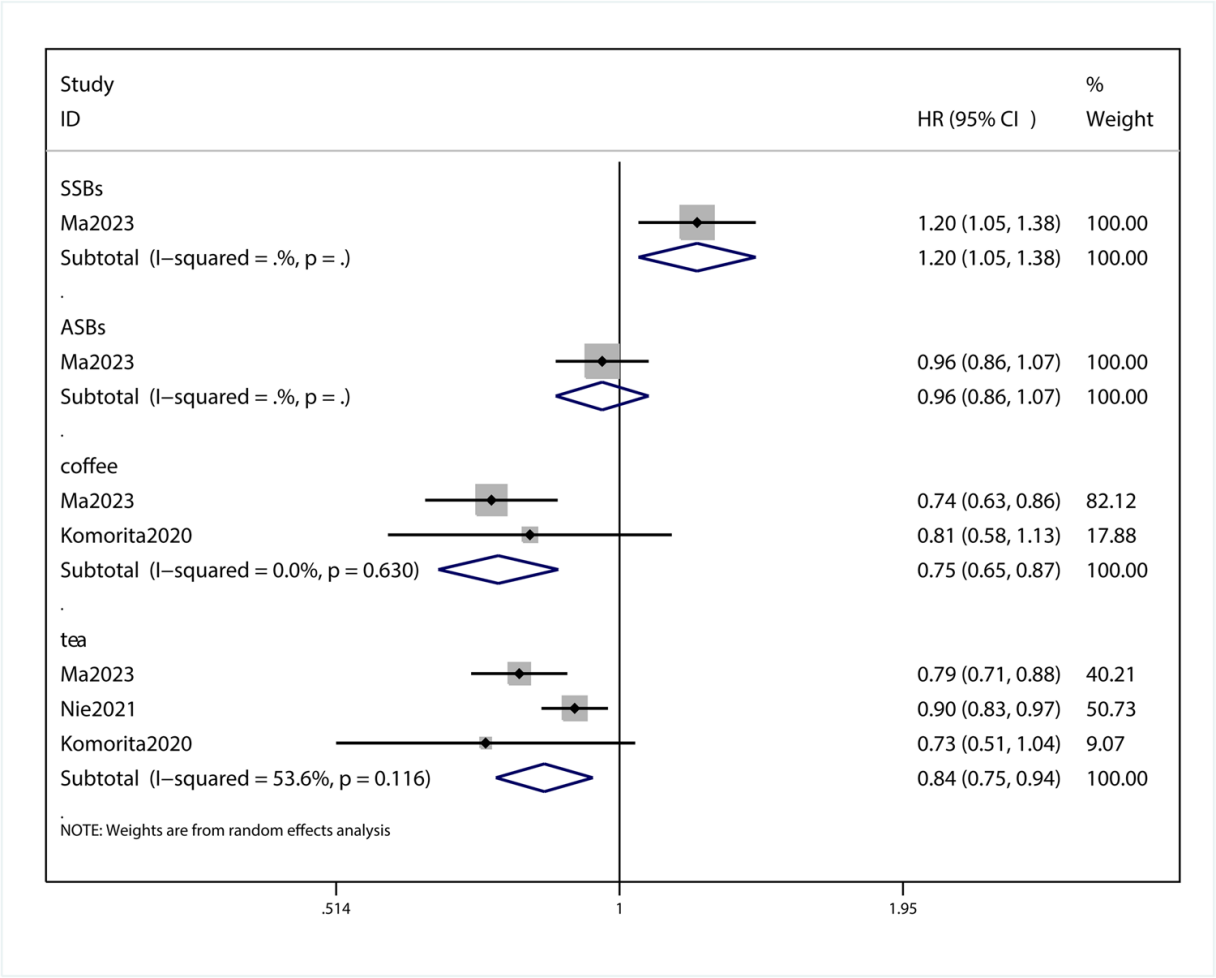
Forest plot of the HR of this meta-analysis for the all-cause mortality in patients with T2DM intaking beverages

This meta-analysis with 3 observational studies was designed to investigate the association between beverage consumption and T2DM mortality. A meta-regression was conducted to calculate the P value for the heterogeneity between groups**,** between-study heterogeneity was quantified by I^2^ statistic(I^2^ = 80.7%, *p* = 0.046). *P* < 0.1 was considered statistically significant for the Q statistic and I^2^ values of approximately 80.7% were considered high heterogeneity. A subgroup analysis was conducted to detect potential sources of heterogeneity. After the data extraction and comprehensive analysis of the selected research articles, we obtained HR of 1.20 (95%Cl,1.05–1.38; *P* = 0.01; Fig. 2) for SSB, 0.96 (95%CI, 0.86–1.07; *P* = 0.464; Fig. 2) for ASB,0.75 (95% CI, 0.65–0.87; *P* < 0.001; Fig. 2) for coffee, 0.84 (95% Cl, 0.75–0.94; *P* = 0.002; Fig. 2) for tea. From subgroup analysis, this group of data shows that coffee and tea can reduce the mortality of T2DM, and ASBs can reduce the mortality of T2DM. No obvious impact, SSBs can lead to increased mortality of T2DM. Sensitivity analysis showed that omitting each study did not change the significance of the results (SFigure 1). The funnel plot was visually symmetrical (SFigure 2), and Begg test showed a none significant publication bias (*P* = 0.679; SFigure 3).

### Association of intaking beverages and incidence risk for T2DM

**Fig. 3:**
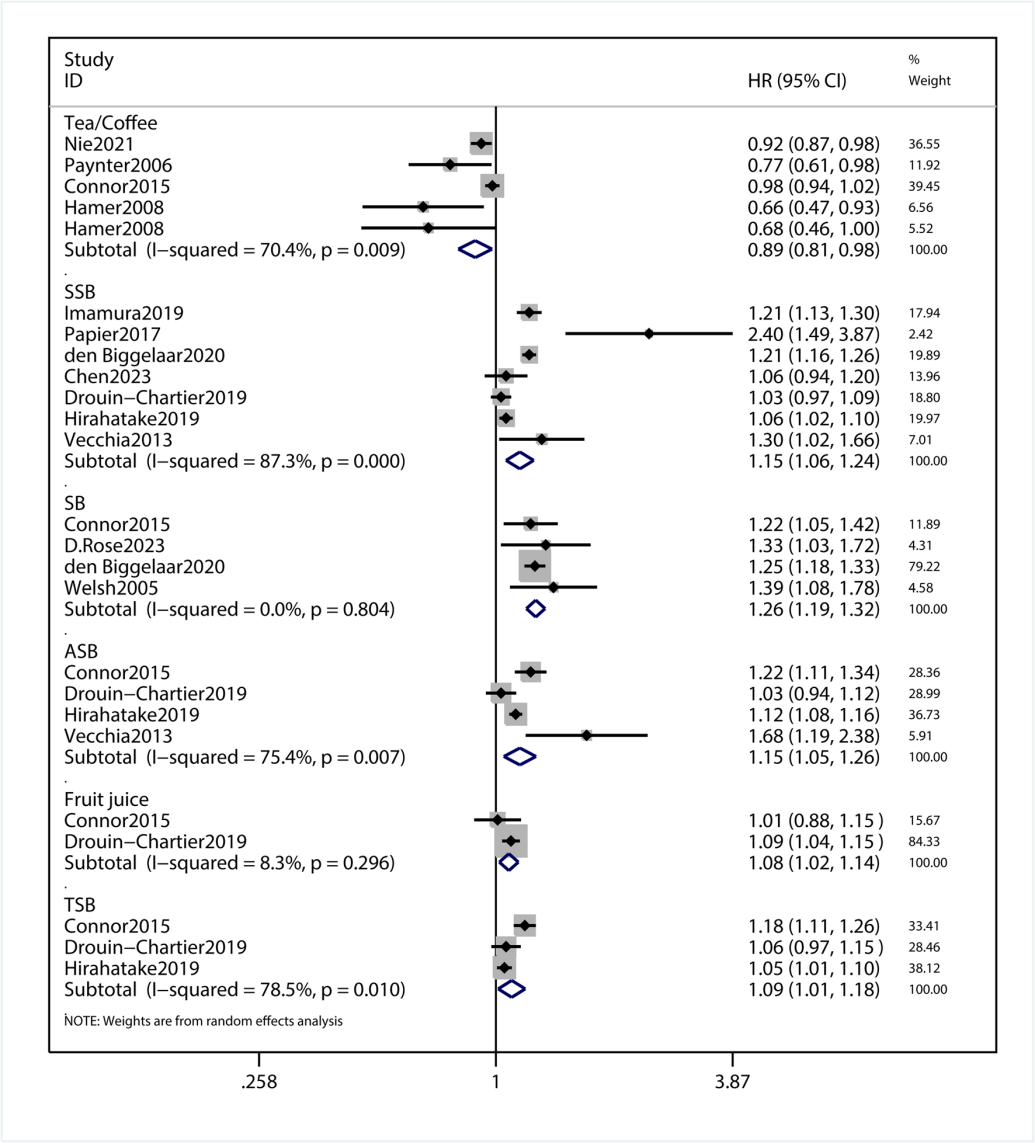
Forest plot of the HR of this meta-analysis for the incidence risk of T2DM in patients intaking beverages

This meta-analysis with 13 observational studies was designed to investigate the association between beverage consumption and T2DM risk. In this meta-analysis, we pooled the data for analysis the association of beverages and incidence risk for T2DM. Between-study heterogeneity was quantified by I^2^ statistic(I^2^ = 87.2%, *p* = 0.000). *P* < 0.1 was considered statistically significant for the Q statistic and I^2^ values of approximately 87.2% were considered high heterogeneity. A subgroup analysis was conducted to detect potential sources of heterogeneity. The results showed that HR was 0.89 (95%CI,0.81–0.98; *P* = 0.016; Fig. 3) for tea or coffee, 1.15 (95% CI,1.06–1.24; *P* = 0.001; Fig. 3) for SSB, 1.26(95% CI,1.19–1.32,* P* < 0.001; Fig. 3) for SB, 1.15 (95% CI,1.05–1.26, *P* = 0.003) for ASB, 1.08 (95% CI,1.02–1.14; *P* = 0.007; Fig. 3) for fruit justice, 1.09 (95% CI,1.01–1.18; *P* = 0.021; Fig. 3)for total sweetened beverage (TSB; combined ASB and SSB). From the subgroup analysis, it can be concluded that tea or coffee can reduce the incidence of T2DM, SSB, TSB and ASB will increase the incidence of T2DM, while fruit justice and TSB have little impact on the incidence of T2DM. Sensitivity analysis showed that omitting each study did not change the significance of the results (SFigure 4). The funnel plot was visually symmetrical (SFigure 5), and Begg's test showed a no significant publication bias (*P* = 0.676; SFigure 6).

### Association of drinking beverages and cardiovascular complications of T2DM patients

**Fig. 4:**
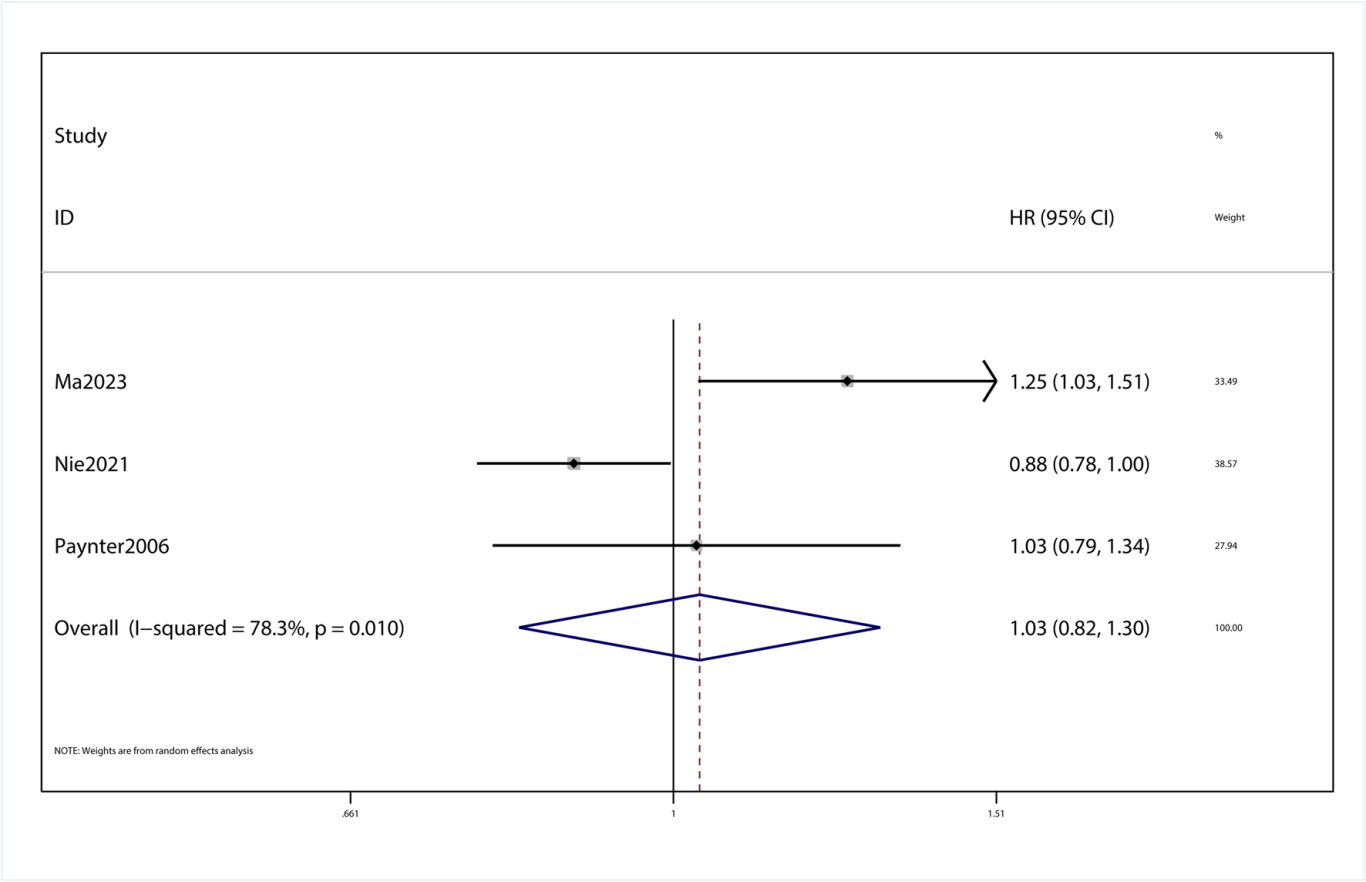
Forest plot of the HR of this meta-analysis for cardiovascular complications in patients with T2DM intaking beverages

This meta-analysis with 3 observational studies was designed to investigate the association between beverage consumption and cardiovascular complications of T2DM patients. Between-study heterogeneity was quantified by I^2^ statistic(I^2^ = 78.3%, *p* = 0.776). *P* = 0.776 wasn’t considered statistically significant for the Q statistic and I^2^ values of approximately 78.3% were considered high heterogeneity. During the process of data extraction, it was found that data related to cardiovascular complications were extracted for only one beverage in one article. In the study of cardiovascular complications, subgroup analysis of cardiovascular complications was not performed in this meta-analysis because the issue of subgroups was not involved in the included articles. In the study of the effects of beverages on cardiovascular complications, we found that the HR was 1.03 (95% Cl, 0.82–1.30, *P* > 0.05; Fig. 4). Sensitivity analysis showed that omitting each study did not change the significance of the results (SFigure 7). The funnel plot was visually symmetrical (SFigure 8), and Begg's test showed a no significant publication bias (*P* = 0.531; SFigure 9).

## Discussion

This present meta-analysis with 15 observational studies was designed to investigate the association between beverage consumption and T2DM risk and mortality, as well as its impact on cardiovascular complications. This is the first paper to synthesize the effects of consumption of SSBs, coffee, and tea on the incidence of T2DM, mortality, and complication of cardiovascular disease. To some extent, the results of this meta-analysis support the hypothesis that SSB consumption was associated with a significant increased overall T2DM risk and mortality, and that tea and coffee intake reduced overall T2DM risk in the study. What's more, a significant dose–response relationship was observed between SSBs or tea and coffee consumption and overall T2DM risk, strengthening this hypothesis. It has guiding significance for the adjustment of diet structure between diabetic patients and normal people, and may have clinical significance in the prevention and prognosis of T2DM.

Beverage consumption has some influence on T2DM incidence and mortality, and research is gradually discovering a variety of other effects. The impact of the intake of the most important SSBs, coffee and tea, on the incidence and mortality of T2DM has attracted extensive attention. Previous studies have reported that consumption of SSBs is associated with an increased risk of T2DM, while replacing SSBs with tea or coffee is associated with a reduced risk of T2DM [5]. There is another study in Chinese adults shows, daily green tea consumption was associated with a lower risk of incident T2D and a lower risk of all-cause mortality in patients with diabetes [6]. Recent study has shown that consuming sugary drinks increases the risk of death from T2DM, whereas intakes of coffee, tea, plain water, and low fat milk were inversely associated with all-cause mortality [7]. In other studies, drinking SSBs, coffee and tea has been found to have a potential impact on the development of cardiovascular disease. Studies have shown that drinking coffee or more tea can reduce the risk of cardiovascular disease and death [21, 22]. But no studies have combined the effects of SSBs, coffee and tea on T2DM and cardiovascular disease. The study was conducted to facilitate follow-up studies on the association between beverage intake and the effects of different diseases.

This study shows that coffee and tea can reduce the mortality of T2DM, ASBs has no significant effect on the mortality of T2DM, and SSBs can increase the mortality of T2DM, which is consistent with the results of previous studies. There is an obvious reduction in mortality risk of T2DM compared with participants who never consumed tea in a previous research [6]. In one study, high consumption of green tea and coffee was associated with reduced all-cause mortality: their combined effect appeared to be additive in people with T2DM. This is consistent with our findings [14]. In another study, increased ASB intake was associated with an increased risk of diabetes. Replacing one daily serving of SSBs with water, coffee or tea reduces diabetes risk by 2–10% [3], which is consistent with our findings. In one study [23], frequent SSB intake was associated with a higher incidence of T2DM in women, which is consistent with our findings.

This study shows that SSBs have less effect on cardiovascular complications. In one study, higher intake of SSBs was associated with cardiovascular morbidity and mortality, while intake of coffee, tea, plain water and low-fat milk was inversely associated with all-cause mortality. This is consistent with our findings, but its clinical value remains to be studied due to the high intake of SSBs and the low correlation with cardiovascular complications [7].

The strength of our study includes the large number of healthy people and cases of T2DM to ensure a greater precision and high statistical power of the results. Our findings provide an assumption that consumption of SSBs should be further considered as a risk factor for T2DM risk and mortality, and that coffee and tea consumption may reduce T2DM risk and mortality. Even so, we have to admit that there are several limitations in our meta-analysis. Firstly, the studies included in the analysis were observational studies, such as case–control and cohort studies, which are more susceptible to bias, such as selection bias and recall bias. Secondly, although the sensitivity analysis showed the stability of these results by omits some studies, differences remained in the potential bias of each study, the definition and scope of beverage consumption, the type of questionnaire, and confounding factors that adjusted the analysis. These differences may all affect the accuracy of these results. In addition, due to insufficient data, this meta-analysis was not carried out dose–response relationship. The last limitation is that the included study populations come from different regions, and people from different regions have specific eating behaviors. As a result, the scope of research in the world is limited due to the small amount of research from Africa and Asia. Therefore, the overall findings of increased incidence and mortality of diabetes should not be overemphasized.

A large number of studies have shown that beverage intake is associated with the occurrence of T2DM, death and cardiovascular disease in the population. The intake of different types of beverages and the difference of intake will have different effects on the incidence, mortality and cardiovascular disease incidence. [24–26]According to research, this may be related to several biological mechanisms. These biological mechanisms may explain the different presumed associations between certain types of beverages and the incidence of T2DM, mortality, and cardiovascular disease in the population. The positive association between consumption of sugary beverages and adverse health outcomes may be related to high glucose and fructose intake. [27, 28]Adding glucose and fructose syrup to sugary drinks can lead to obesity, dyslipidemia, insulin resistance and inflammation [29–31].The consumption of fructose and glucose from sugar-sweetened beverages can lead to incomplete metabolism of calories, adverse glycemic effects, as well as fat reproduction and visceral fat accumulation, which is seriously harmful for type 2 diabetes patients [29].In particular, fructose intake, due to the lack of feedback mechanism, will lead to substrate accumulation, accompanied by increased triglyceride level, increased fat content in hepatocytes and gluconeogenesis, which further increases blood sugar, aggravates the symptoms of T2DM and increases the cardiovascular burden [30].Although the mechanism of diabetes and atherosclerosis is not clear in the current study, clinical and pathological analysis has found that the inflammatory response is more obvious, the hardening process is more active, and the range of sclerosis is more extensive in the atherosclerosis of diabetic patients, which may be related to the production of inflammatory cytokines, oxidative stress, and endothelial dysfunction [32].At the same time, the insulin resistance seen in many people with type 2 diabetes is partly associated with obesity. The possible mechanisms are the effects of fatty acids, inflammatory cytokines, adipokines, and beta cell dysfunction [33].Numerous preclinical and clinical studies have demonstrated a causal relationship between aseptic low-grade inflammation and type 2 diabetes. Chronic inflammation in type 2 diabetes and obesity mainly involves monocytes, usually with a 2–threefold increase in pro-inflammatory cytokines and chemokines, and is not confined to a specific site but distributed throughout the organ system, where activation of the immune system in the islets leads to damage of beta cells [34].Type 2 diabetes is a chronic, progressive disease associated with obesity, fat accumulation, inflammatory cytokine release, and insulin resistance. This process is exacerbated by the consumption of sugary beverages, which increases the incidence of type 2 diabetes, mortality, and cardiovascular complications. In contrast, coffee and tea do not contain large amounts of glucose and fructose. Among them, the bioactive components of coffee include caffeine, chlorogenic acid, trigonelline, tryptophan alkaloids and other secondary metabolites [35].Possible mechanisms by which chlorogenic acid may be beneficial to health include alleviating oxidative stress responses and exhibiting anti-inflammatory activity in some important metabolic pathways [36].Trigonelline also has a protective effect on type 2 diabetes. Trigonelline can significantly reduce the levels of blood glucose, serum tumor necrosis factor-α, interleukin-6 and interleukin-1β in diabetic mice, and increase the levels of serum insulin and adiponectin [37].Among the many bioactive compounds also contained in tea, the main antioxidant is catechins, which also have anti-inflammatory and anticancer effects. Catechins have a strong ability to neutralize reactive oxygen species and active nitrogen [38, 39]. Green tea catechin derivatives include: epicatechin, epigallocatechin, epicatechin gallate, and epigallocatechin gallate, the last of which has the most potent anti-inflammatory and anticancer potential [38].In oral cancer, tea intake has been shown to have a protective effect [40].Epigallocatechin gallate inhibits cytokine induced pancreatic beta cell damage. EGCG induced significant decreases in IL-1b and IFN-γ-induced nitric oxide production, and decreased levels of the inducible form of NO synthase (iNOS) mRNA and protein on RINm5F cells, suggesting that EGCG intake may help alleviate the symptoms of type 2 diabetes [41].Low-fat milk and whole milk do not show a harmful association with cardiovascular disease or type 2 diabetes in most observational and experimental evidence. And milk contains a variety of bioactive substances that may help improve the host and microbiome in the gastrointestinal environment. [42].

## Conclusions

In summary, our findings suggest that there is a positive association between SSBs consumption and overall T2DM risk and mortality, while there is a negative association between coffee and tea consumption and overall T2DM risk and mortality, although the evidence is limited. More large and precise prospective studies are required to further assess the association and the underlying mechanisms between them.

### Supplementary Information

Below is the link to the electronic supplementary material.

Supplementary file1 **SFigure 1.** Sensitivity analysis showed that the overall pooled results of all-cause mortality was robust and reliable about the intaking beverages and patients with T2DM. (PNG 87 kb)

High resolution image (TIF 1280 kb)

Supplementary file2 **SFigure 2.** Funnel plot showed the all-cause mortality of intaking beverages in patients with T2DM. (PNG 67 kb)

High resolution image (TIF 1024 kb)

Supplementary file3 **SFigure 3.** Publication bias of the overall pooled all-cause mortality showed no potential publication bias in this meta-analysis. (PNG 62 kb)

High resolution image (TIF 896 kb)

Supplementary file4 **SFigure 4.** Sensitivity analysis showed that the overall pooled results of incidence of T2DM was robust and reliable about the intaking beverages patients and no intaking patients. (PNG 198 kb)

High resolution image (TIF 2195 kb)

Supplementary file5 **SFigure 5.** Funnel plot showed the incidence of T2DM between intaking beverages and no intaking beverages group. (PNG 76 kb)

High resolution image (TIF 1053 kb)

Supplementary file6 **SFigure 6.** Publication bias of the overall pooled results of incidence showed no potential publication bias in this meta-analysis. (PNG 58 kb)

High resolution image (TIF 905 kb)

Supplementary file7 (PNG 76 kb)

High resolution image (TIF 121 kb)

Supplementary file8 (PNG 172 kb)

High resolution image (TIF 334 kb)

Supplementary file9 (PNG 76 kb)

High resolution image (TIF 121 kb)
