# Supplementary figures and images for: Effects of sugary drinks, coffee, tea and fruit juice on incidence rate, mortality and cardiovascular complications of type2 diabetes patients: a systematic review and meta-analysis
Source: J Diabetes Metab Disord. 2024 Apr 8;23(1):1113–23. doi: 10.1007/s40200-024-01396-5 (PMC11196440; doi:10.1007/s40200-024-01396-5)

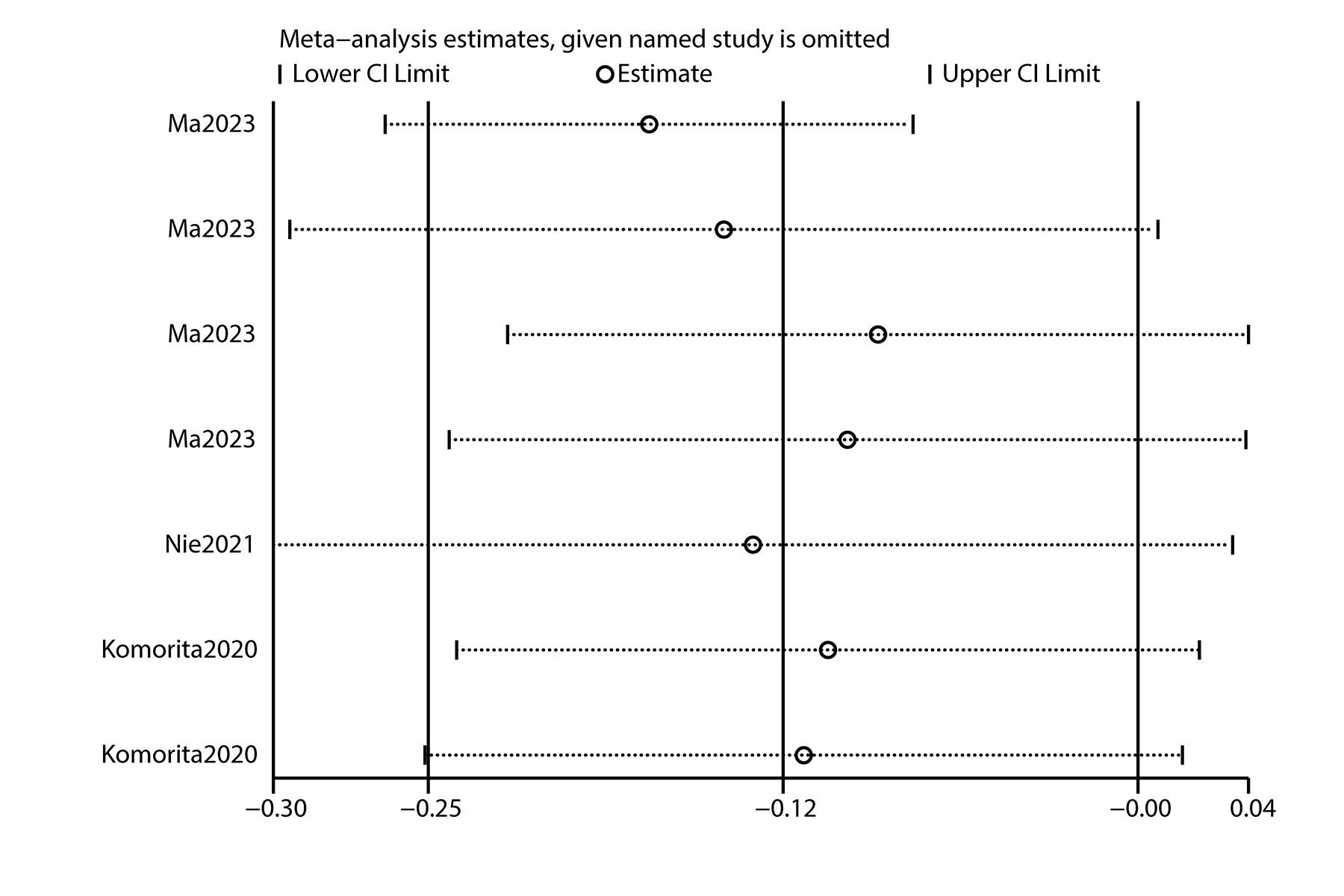

Supplement: Supplementary file 1 — Supplementary file1 SFigure 1. Sensitivity analysis showed that the overall pooled results of all-cause mortality was robust and reliable about the intaking beverages and patients with T2DM. (PNG 87 kb) [file 40200_2024_1396_Fig5_ESM.png]

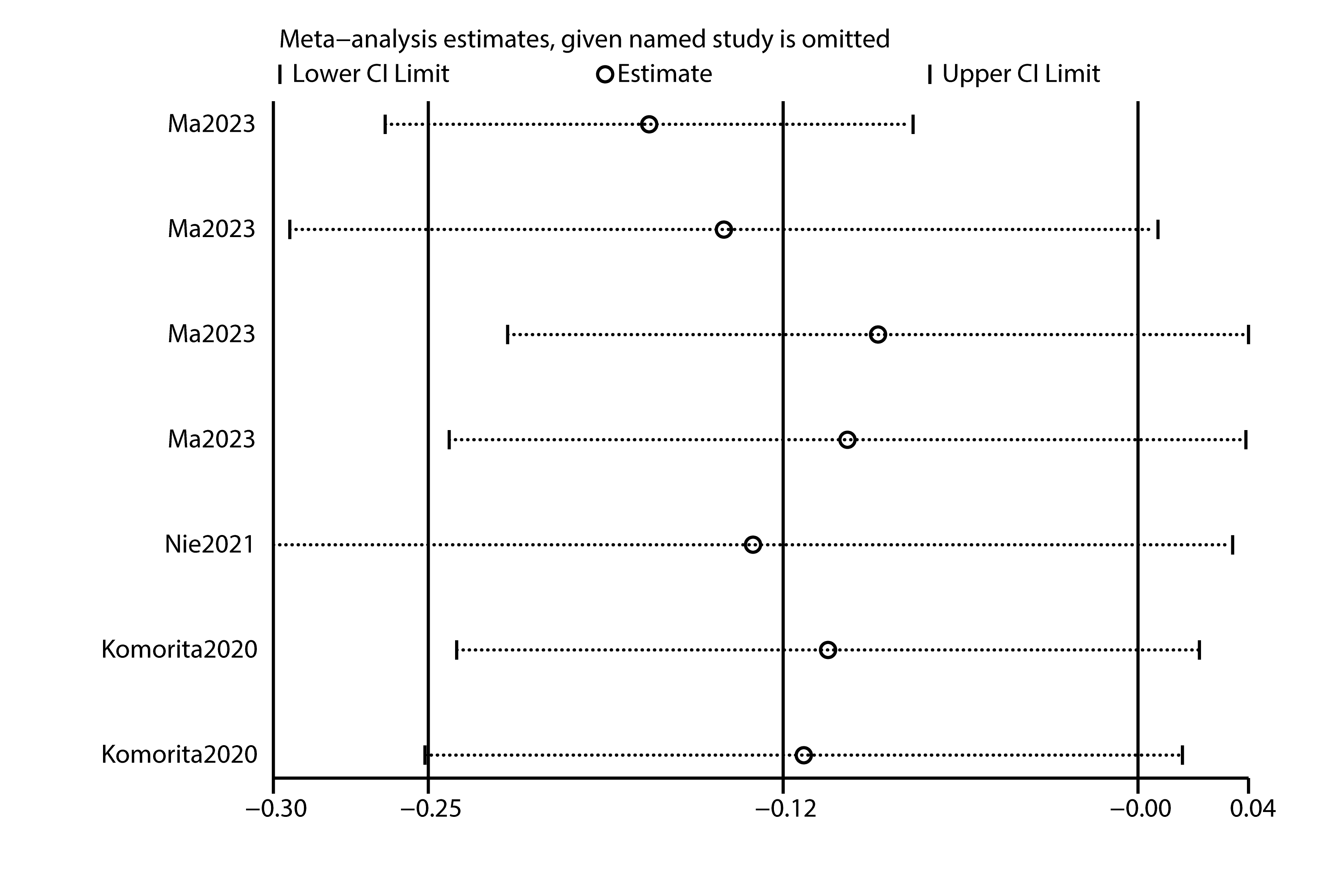

Supplement: Supplementary file 2 — High resolution image (TIF 1280 kb) [file 40200_2024_1396_MOESM2_ESM.tif]

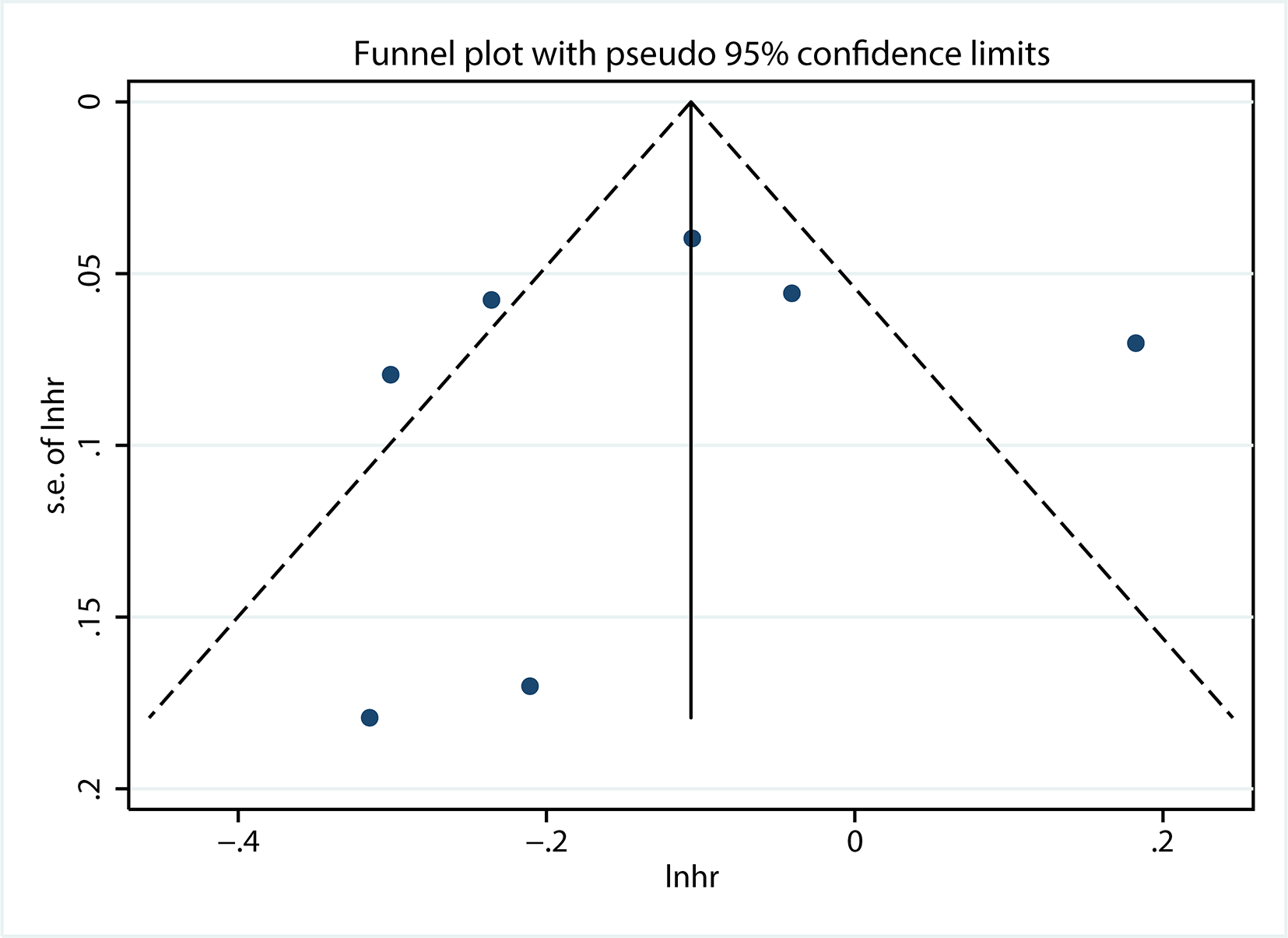

Supplement: Supplementary file 3 — Supplementary file2 SFigure 2. Funnel plot showed the all-cause mortality of intaking beverages in patients with T2DM. (PNG 67 kb) [file 40200_2024_1396_Fig6_ESM.png]

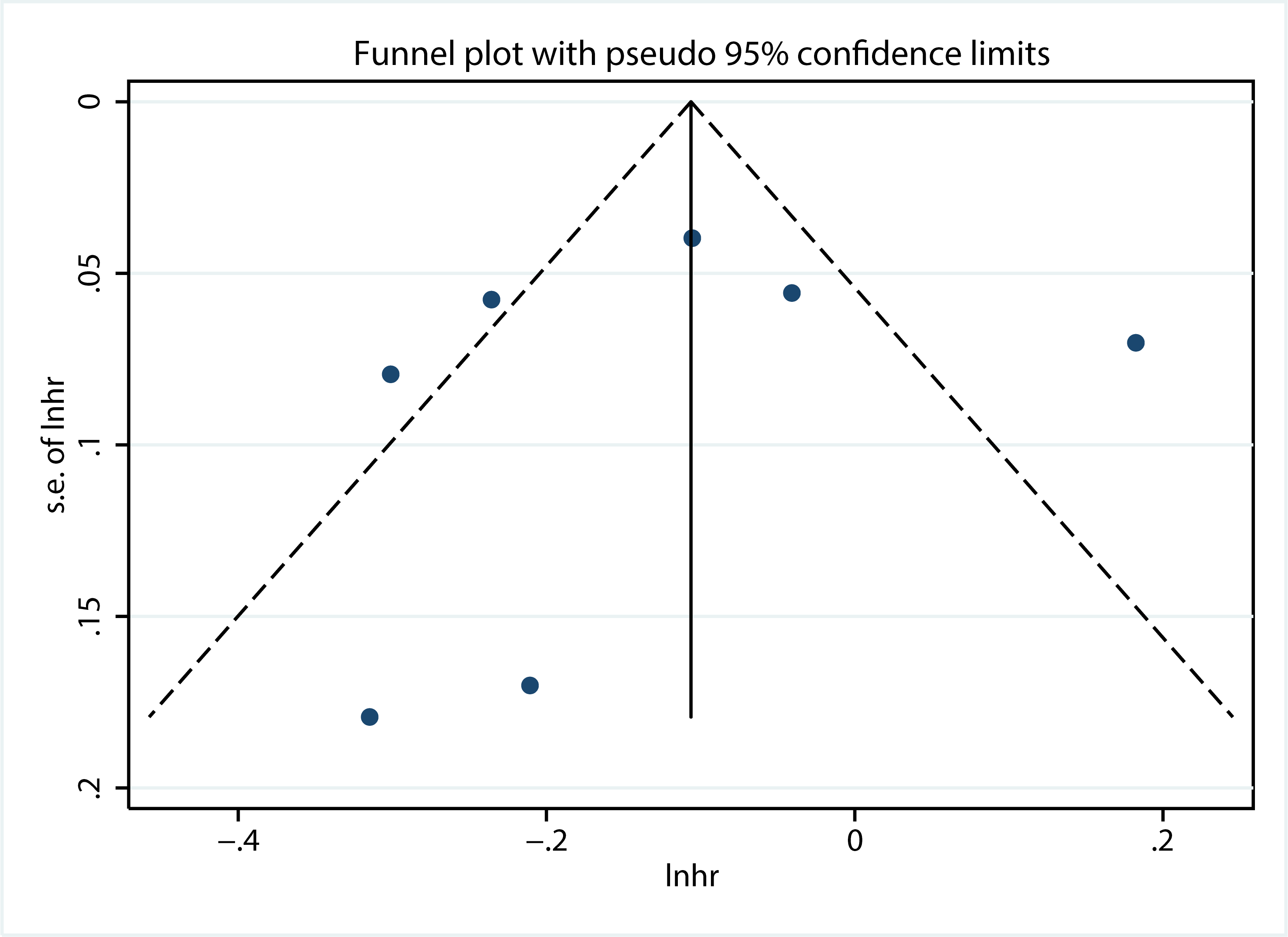

Supplement: Supplementary file 4 — High resolution image (TIF 1024 kb) [file 40200_2024_1396_MOESM4_ESM.tif]

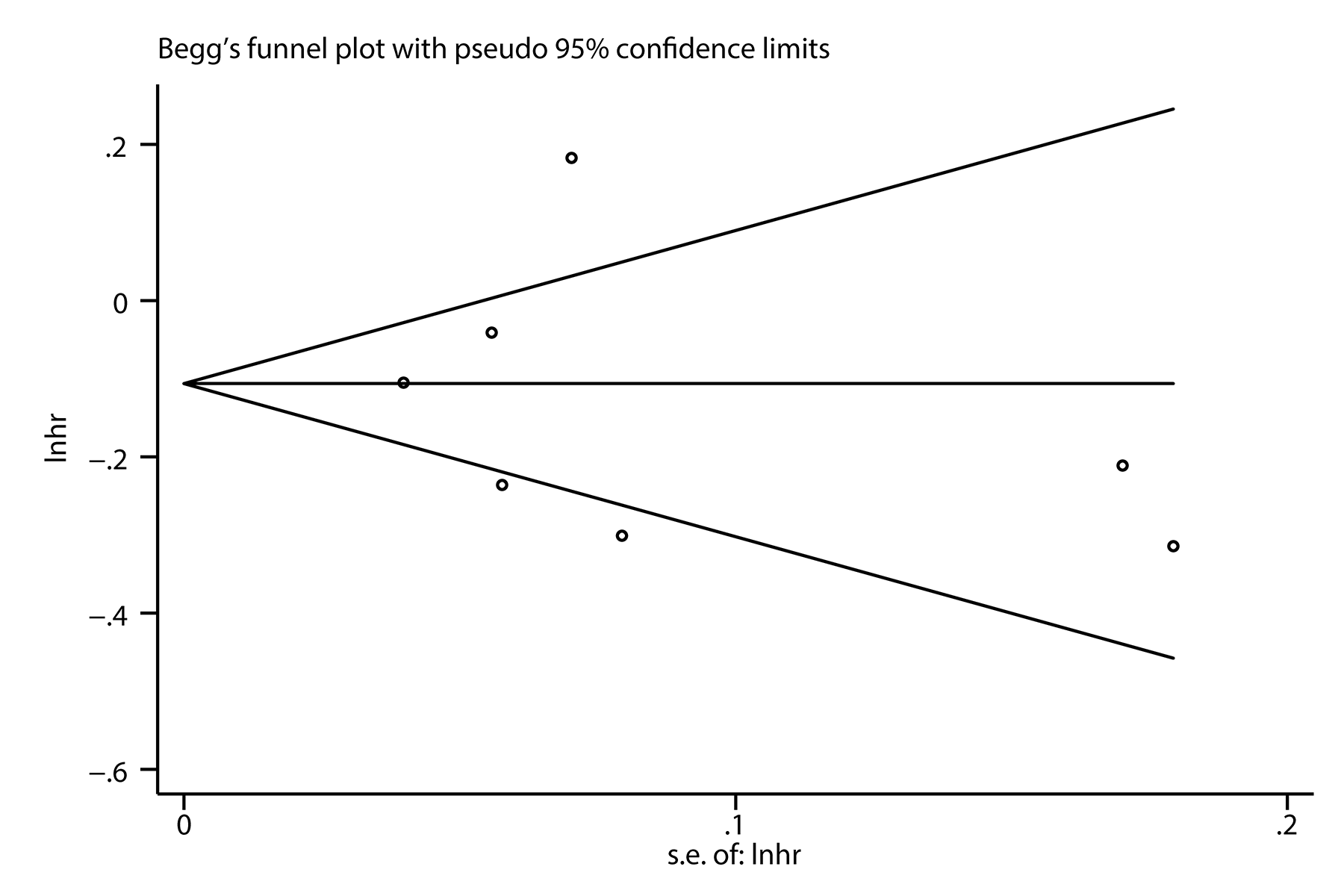

Supplement: Supplementary file 5 — Supplementary file3 SFigure 3. Publication bias of the overall pooled all-cause mortality showed no potential publication bias in this meta-analysis. (PNG 62 kb) [file 40200_2024_1396_Fig7_ESM.png]

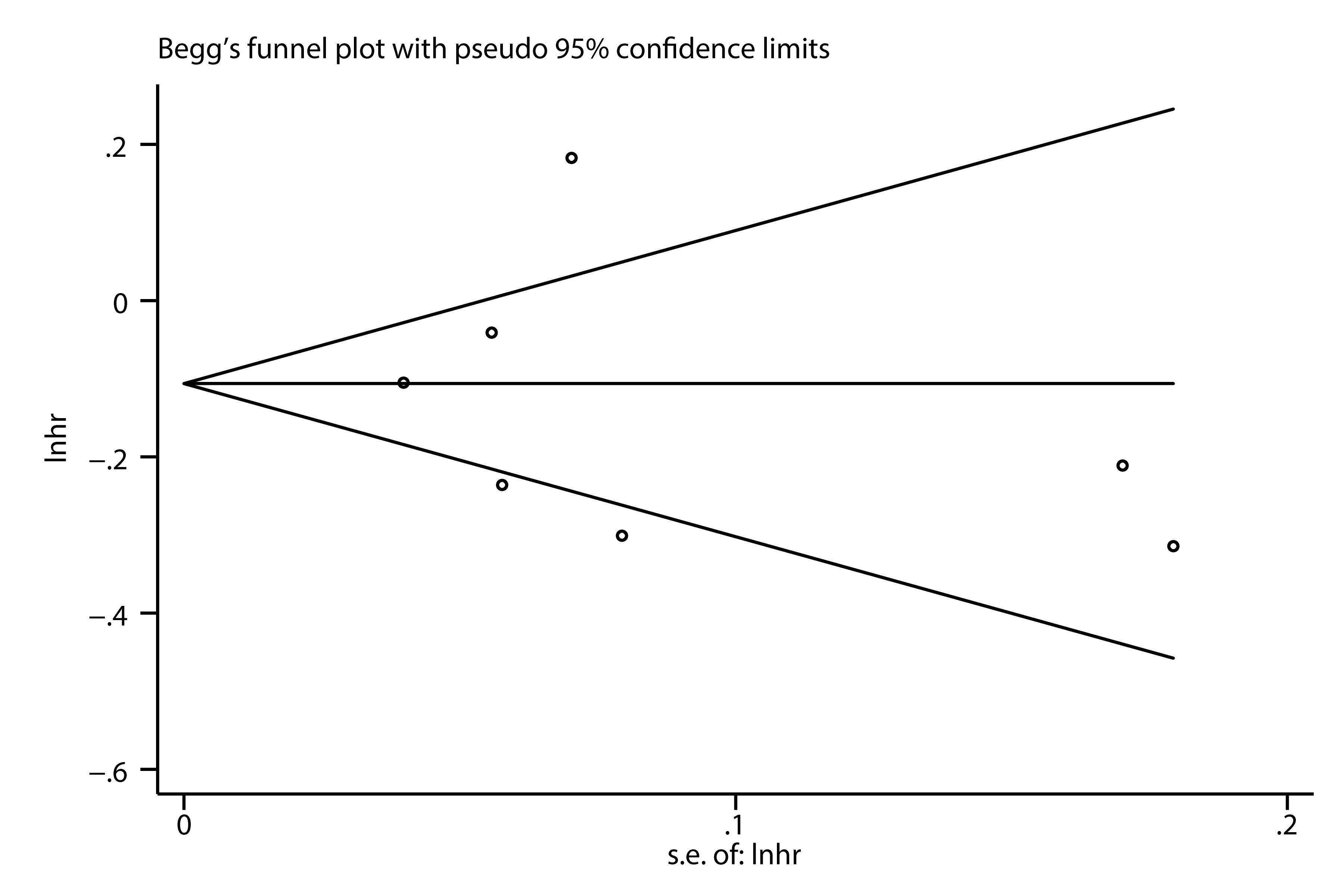

Supplement: Supplementary file 6 — High resolution image (TIF 896 kb) [file 40200_2024_1396_MOESM6_ESM.tif]

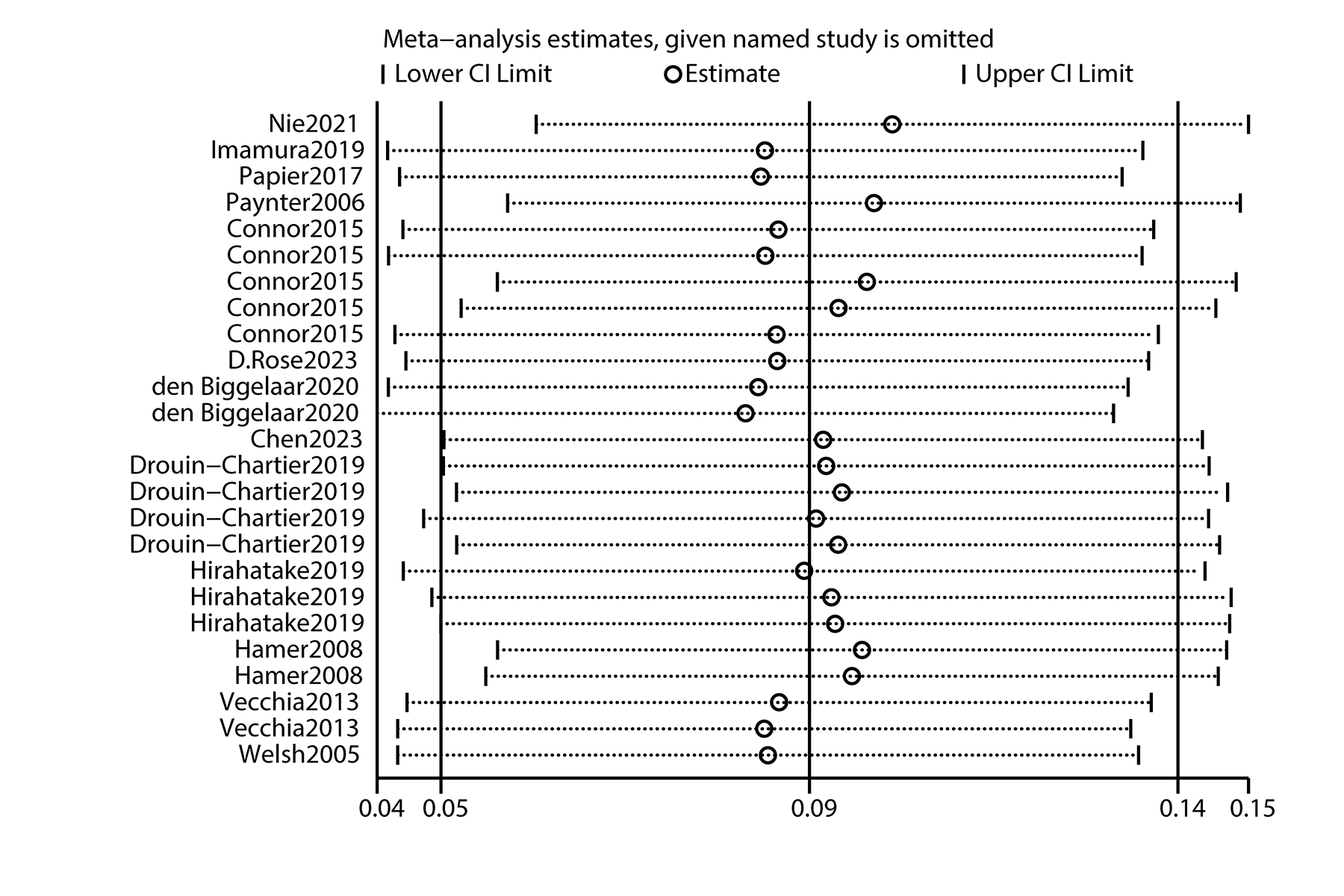

Supplement: Supplementary file 7 — Supplementary file4 SFigure 4. Sensitivity analysis showed that the overall pooled results of incidence of T2DM was robust and reliable about the intaking beverages patients and no intaking patients. (PNG 198 kb) [file 40200_2024_1396_Fig8_ESM.png]

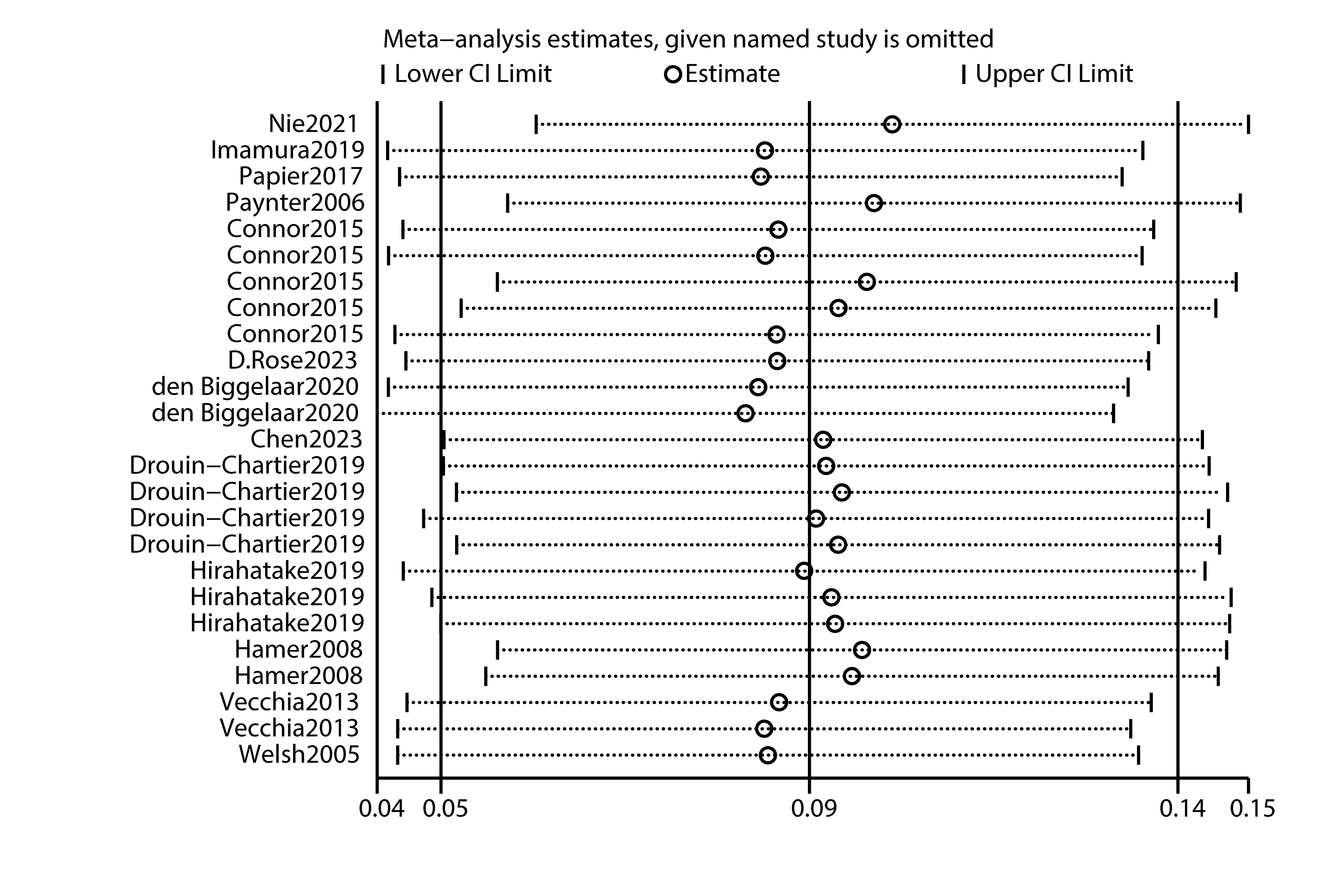

Supplement: Supplementary file 8 — High resolution image (TIF 2195 kb) [file 40200_2024_1396_MOESM8_ESM.tif]

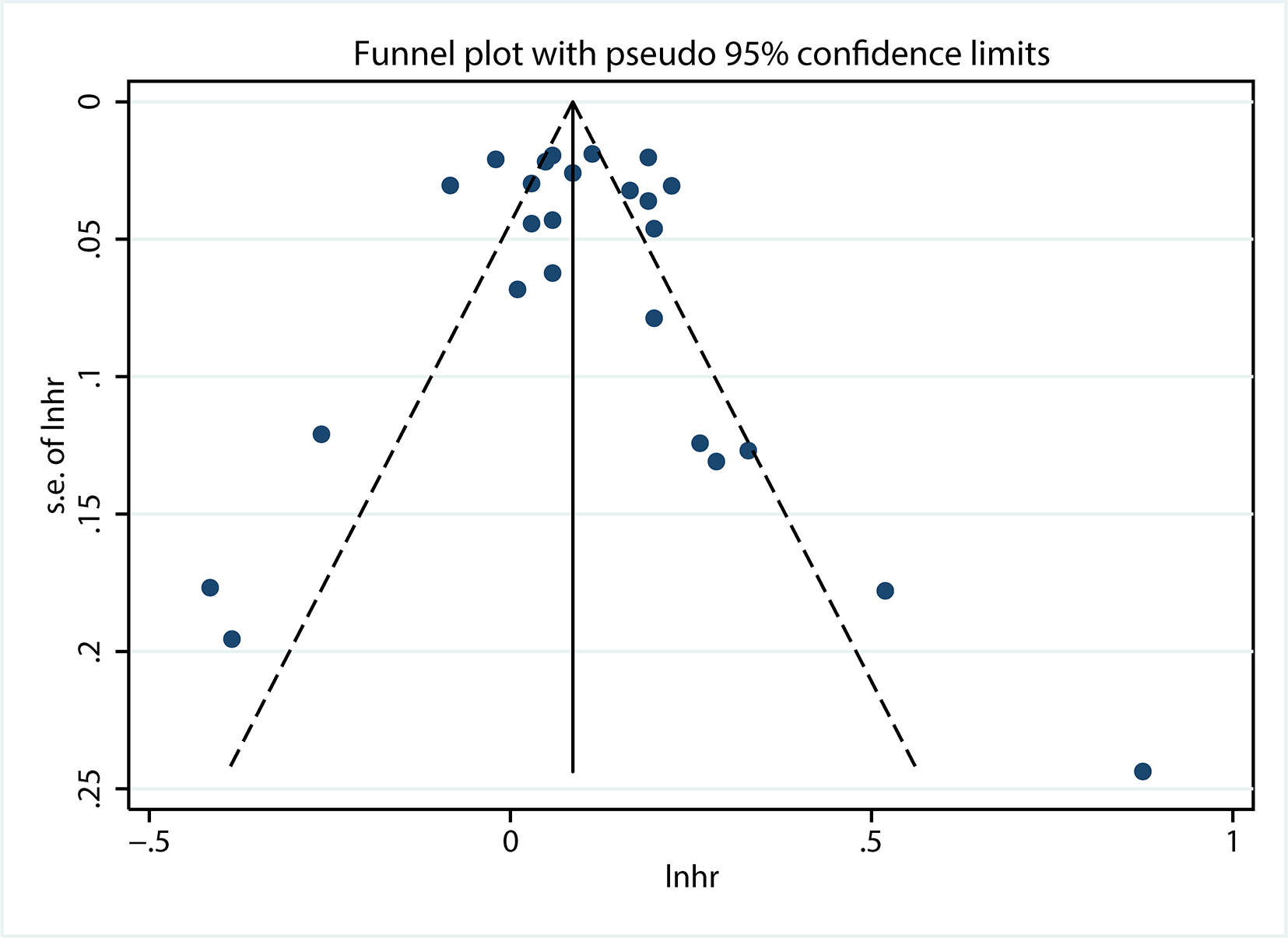

Supplement: Supplementary file 9 — Supplementary file5 SFigure 5. Funnel plot showed the incidence of T2DM between intaking beverages and no intaking beverages group. (PNG 76 kb) [file 40200_2024_1396_Fig9_ESM.png]

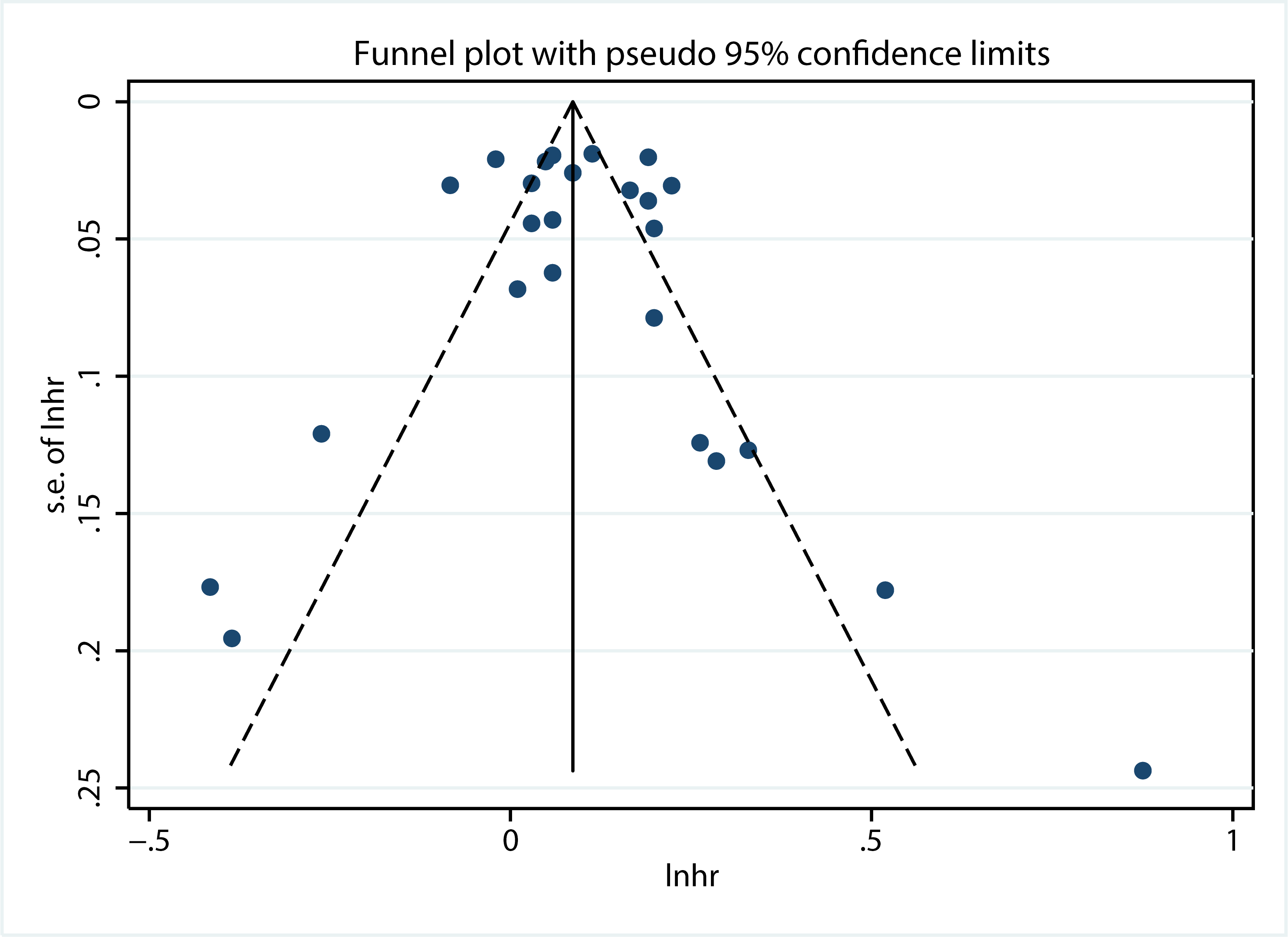

Supplement: Supplementary file 10 — High resolution image (TIF 1053 kb) [file 40200_2024_1396_MOESM10_ESM.tif]

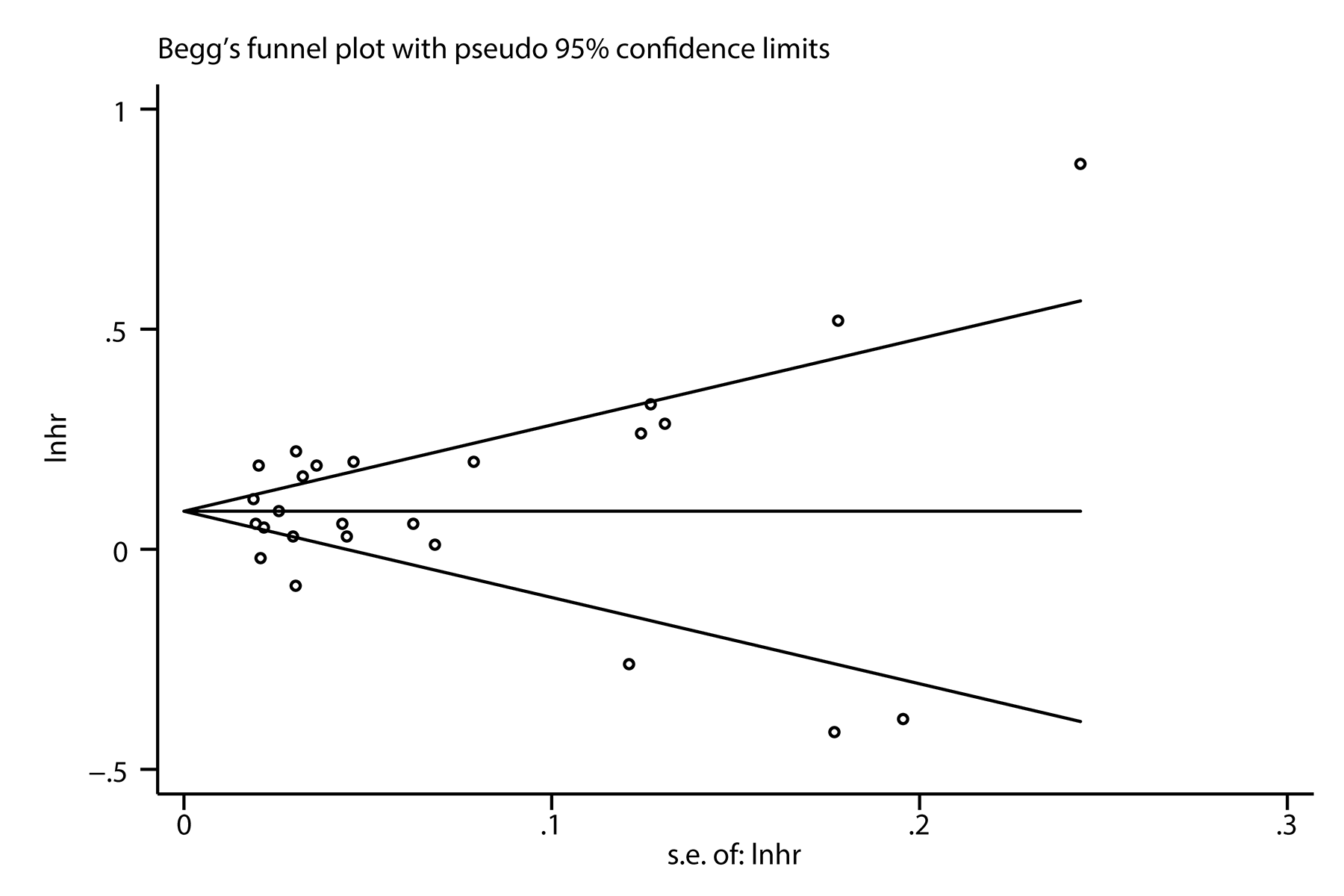

Supplement: Supplementary file 11 — Supplementary file6 SFigure 6. Publication bias of the overall pooled results of incidence showed no potential publication bias in this meta-analysis. (PNG 58 kb) [file 40200_2024_1396_Fig10_ESM.png]

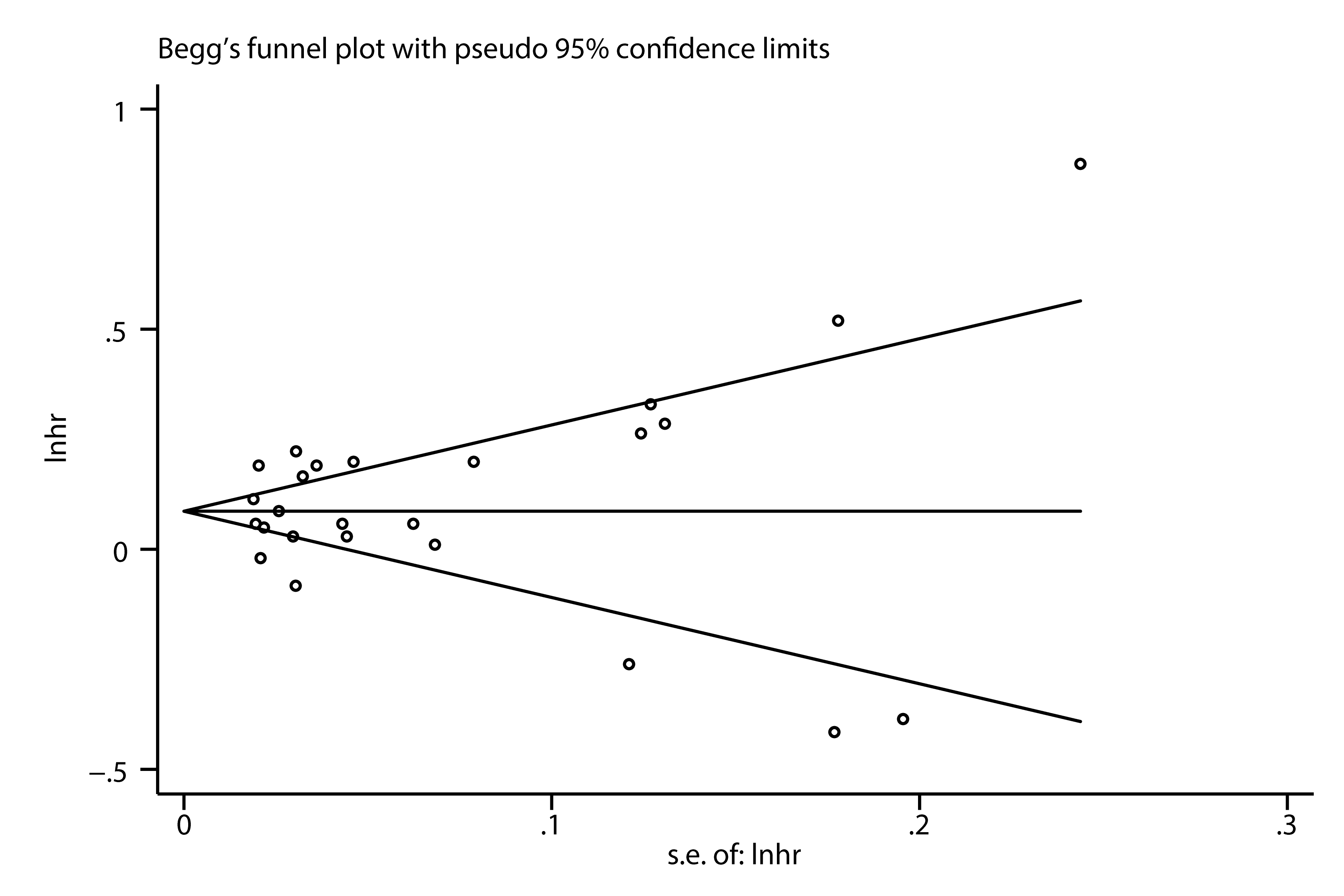

Supplement: Supplementary file 12 — High resolution image (TIF 905 kb) [file 40200_2024_1396_MOESM12_ESM.tif]

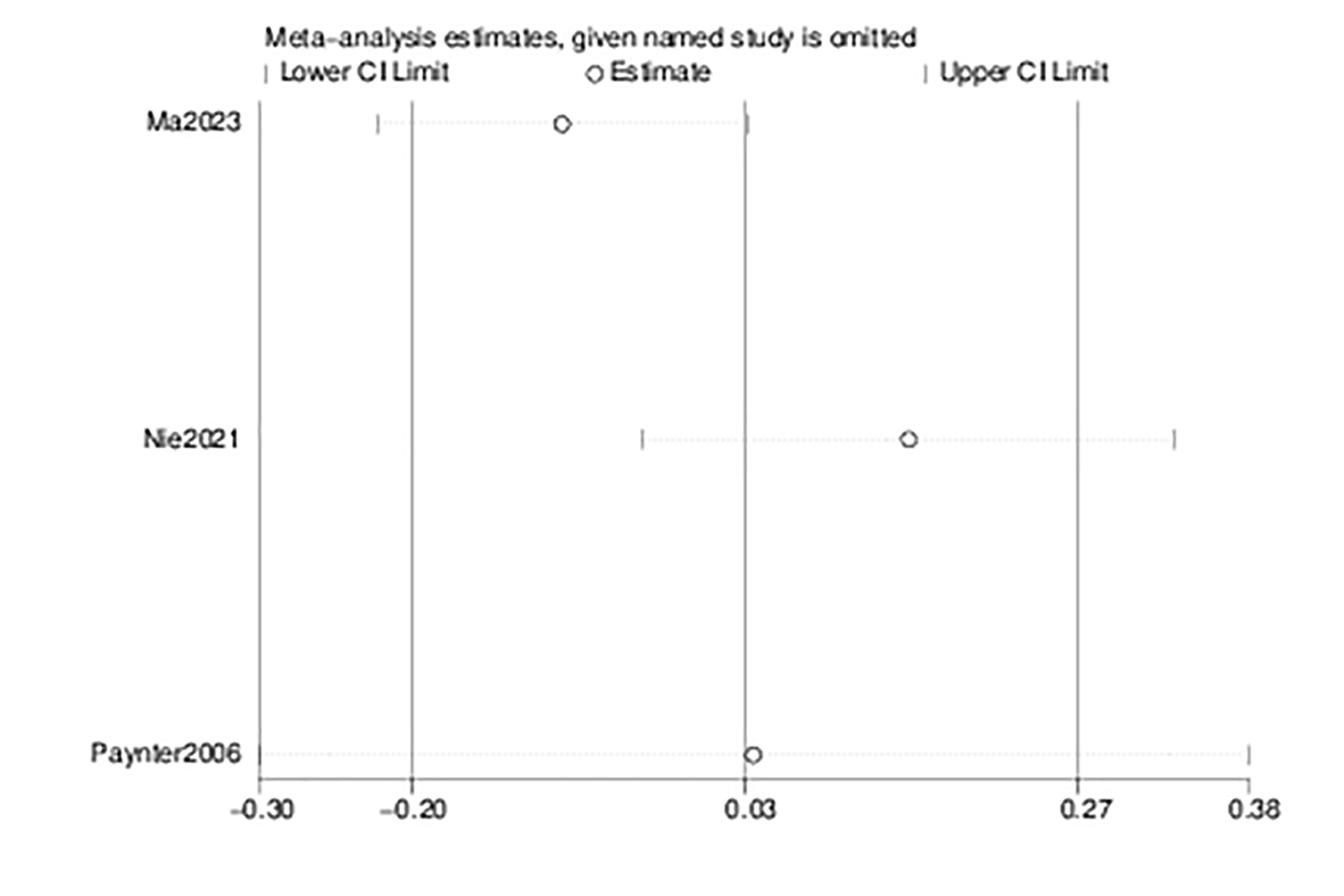

Supplement: Supplementary file 13 — Supplementary file7 (PNG 76 kb) [file 40200_2024_1396_Fig11_ESM.png]

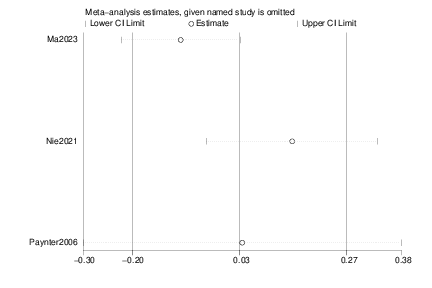

Supplement: Supplementary file 14 — High resolution image (TIF 121 kb) [file 40200_2024_1396_MOESM14_ESM.tif]

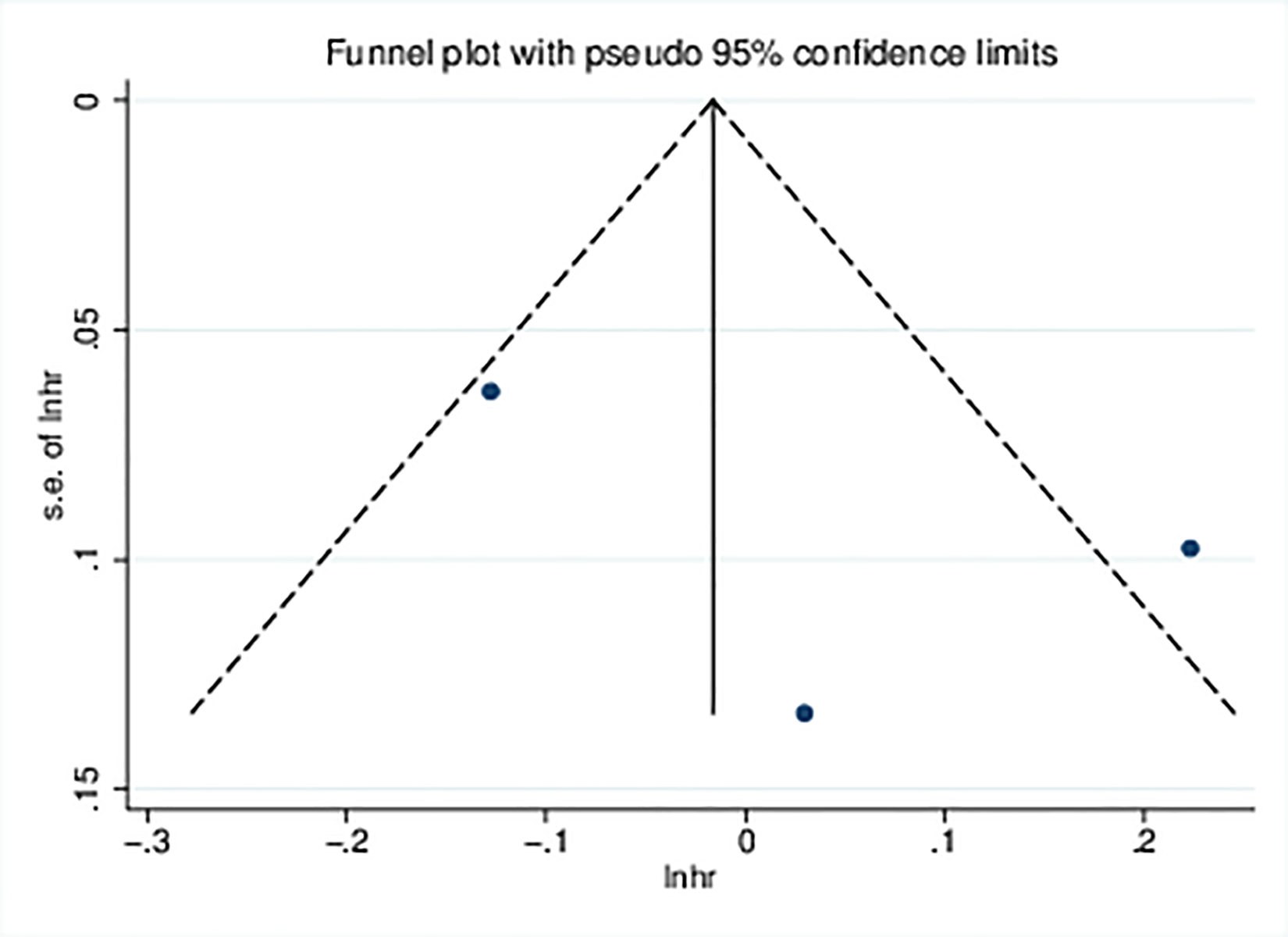

Supplement: Supplementary file 15 — Supplementary file8 (PNG 172 kb) [file 40200_2024_1396_Fig12_ESM.png]

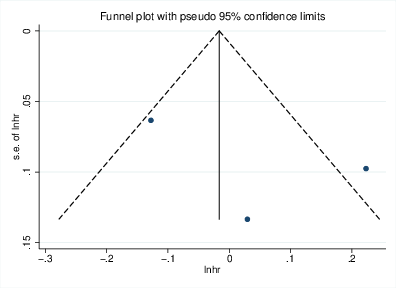

Supplement: Supplementary file 16 — High resolution image (TIF 334 kb) [file 40200_2024_1396_MOESM16_ESM.tif]

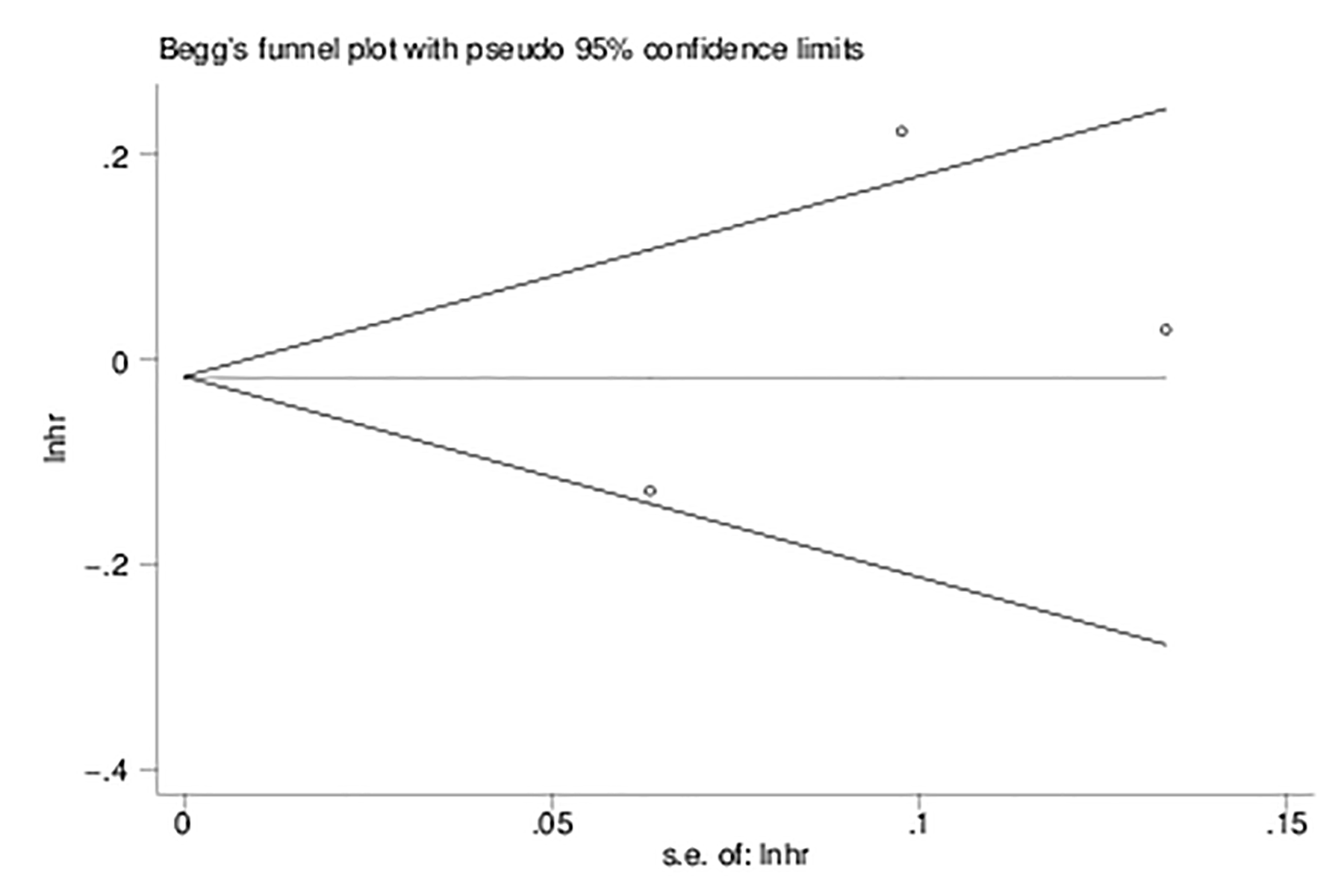

Supplement: Supplementary file 17 — Supplementary file9 (PNG 76 kb) [file 40200_2024_1396_Fig13_ESM.png]

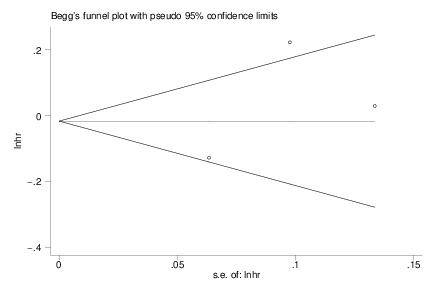

Supplement: Supplementary file 18 — High resolution image (TIF 121 kb) [file 40200_2024_1396_MOESM18_ESM.tif]
